# Comparative Analysis of Aptamer-Conjugated Chemical and Green Synthesized Gold Nanoparticles for Targeted Therapy in MCF-7 Cancer Cells

**DOI:** 10.1007/s12010-024-05091-2

**Published:** 2024-11-27

**Authors:** Mariam W. Helal, Mohanad M. Faried, Sohaila Mohammed Salah, Mazen Ashraf, Nada Nasser, Yasser Shawky, Sara Hamdy, Azza El Amir, Wajeet Nabil, Dalia M. El-Husseini

**Affiliations:** 1 Biotechnology Department, Faculty of Science, Cairo University, Giza, Egypt; 2 Zoology Department, Faculty of Science, Cairo University, Giza, Egypt; 3 Nanomaterials Research and Synthesis Unit, Animal Health Research Institute (AHRI), Agricultural Research Center (ARC), Giza, Egypt

**Keywords:** Aptamer, Breast cancer, Gold nanoparticles, Therapy, Green synthesis

## Abstract

Breast cancer remains a challenging health issue, demanding innovative treatment approaches that maximize efficacy while minimizing damage to healthy cells. Targeted therapy offers a promising strategy tailored to the unique characteristics of breast cancer tumors. Gold nanoparticles have been studied in the context of their therapeutic potential towards cancer treatment showing great success. Recently, aptamers were also investigated for their targeting efficiency towards specific receptors allowing their use in targeting delivery systems. In this study, computational analysis was used to confirm the strong binding between AS1411 aptamer and the nucleolin receptor extensively present on the surface of breast cancer cells, highlighting the aptamer’s potential for specific targeting. Furthermore, we investigated and compared the use of AS1411 aptamer-conjugated chemically synthesized (GNPs) and flaxseed-green-synthesized (Fs-GNPs) gold nanoparticles as targeting therapeutic systems for breast cancer cells. Our results showed successful conjugation of the AS1411 aptamer with both, the GNPs and Fs-GNPs. Characterization of the nanoparticles and their conjugates validates their size, charge, and morphology, affirming the success of the conjugation process. Cytotoxicity assessments using the MTT assay demonstrated the effectiveness of the conjugates against breast cancer cells, with the AS1411-Fs-GNPs conjugate exhibiting higher inhibitory efficacy, featuring an IC_50_ value of 11.13 µg/ml. In contrast, they showed minimal effect on normal cells, emphasizing the selectivity and potential safety of these therapies. To our knowledge, this is the first report of conjugating AS1411 aptamer to green-synthesized gold nanoparticles and its use as a targeting therapeutic system.

**Supplementary Information:**

The online version contains supplementary material available at 10.1007/s12010-024-05091-2.

## Introduction

The crucial purpose of targeting cancer therapeutics is achieving precision and selective treatment to eliminate the threat while preserving normal tissue integrity [1, 2]. Recent years have detected an insightful shift in cancer therapy with targeted therapeutic systems. Integrating these targeted systems into cancer treatment represents a fundamental revolution in the medical approach. This approach promises improved therapeutic outcomes and reduced treatment-related side effects [3], particularly by employing biological agents that play a pioneering role in this innovative landscape [4]. Monoclonal antibodies, aptamers (DNA-RNA), nanoparticles, peptides, viral vectors, RNA interference (RNAi), and CAR T-cell therapy [5] have all played vital roles in customizing treatments for different diseases, especially cancer. Together, these biological agents mark a promising era in precision medicine. Among different strategies, using aptamers has emerged as a promising biological molecule in precision medicine, especially for breast cancer [6].

Breast cancer is a significant cause of female mortality, and finding effective strategies to fight it requires innovative approaches that enhance treatment efficacy while minimizing the adverse effects experienced by patients. Tailoring treatments to the specific characteristics of breast cancer cells is essential [7]. To address this challenge, targeted therapy which utilizes the unique molecular features of cancer cells to precisely target and distinguish between healthy and cancerous cells was investigated [1].

Aptamers, often termed “chemical antibodies,” are single-stranded nucleic acid oligonucleotides renowned for their exceptional specificity and high affinity in binding to specific targets [8]. These molecules are typically discovered through the iterative in vitro process known as the systematic evolution of ligands by exponential enrichment. Aptamers exhibit functional parallels to traditional antibodies but bring forth a range of unique advantages, such as rapid chemical synthesis, adaptability to versatile chemical modifications, outstanding stability, and minimal immunogenicity [6]. Given their remarkable attributes, aptamers have recently emerged as a promising class of agents for the targeted delivery of therapeutic drugs to breast cancer cells, capitalizing on specific cancer-associated hallmarks. This transformative potential places aptamers at the forefront of precision medicine in breast cancer therapy [9]. In recent years, a particular aptamer, AS1411, a 26-base guanine-rich aptamer, adopts a distinctive G-quadruplex structure that exhibits high affinity and specificity for the overexpressed nucleolin receptor (NCL) on the surface of breast cancer cells [10]. However, its impact extends beyond surface binding [11, 12]. Recent studies showed that AS1411, upon internalization by cancer cells, disrupts intracellular activities by forming complexes with NCL and nuclear factor B [13, 14], thus making it a precise targeting and therapeutic molecule [10].

In parallel, GNPs have garnered significant attention as versatile therapeutic agents in recent years. Their unique physical and chemical properties, including tunable size and surface properties, make them amenable to various applications, from drug delivery to imaging [15] GNPs’ intrinsic cytotoxicity is a cornerstone of their appeal in cancer therapy, stemming from their ability to generate reactive oxygen species (ROS) within biological systems [16, 17]. Consequently, oxidative stress can prompt apoptosis or necrosis in cancer cells, ultimately enhancing their death [17]. Eco-friendly green synthesis methods have arisen as pivotal allies to optimize and enhance the therapeutic potential of GNPs [18]. GNPs synthesized via green methods often incorporate natural bioactive components from the reducing agents, enhancing their biocompatibility [19]. Flaxseeds are rich in bioactive compounds and antioxidants [20]. Moreover, the flaxseed-derived compounds within nanoparticles enhance their cytotoxicity against breast cancer cells, offering a novel therapeutic pathway [21]. The conjugation of aptamers to GNPs could enhance their stability as well as their therapeutic efficacy by enabling the selective delivery of drugs or therapeutic payloads directly to cancer cells while minimizing collateral damage to healthy ones [22].

**Fig. 1:**
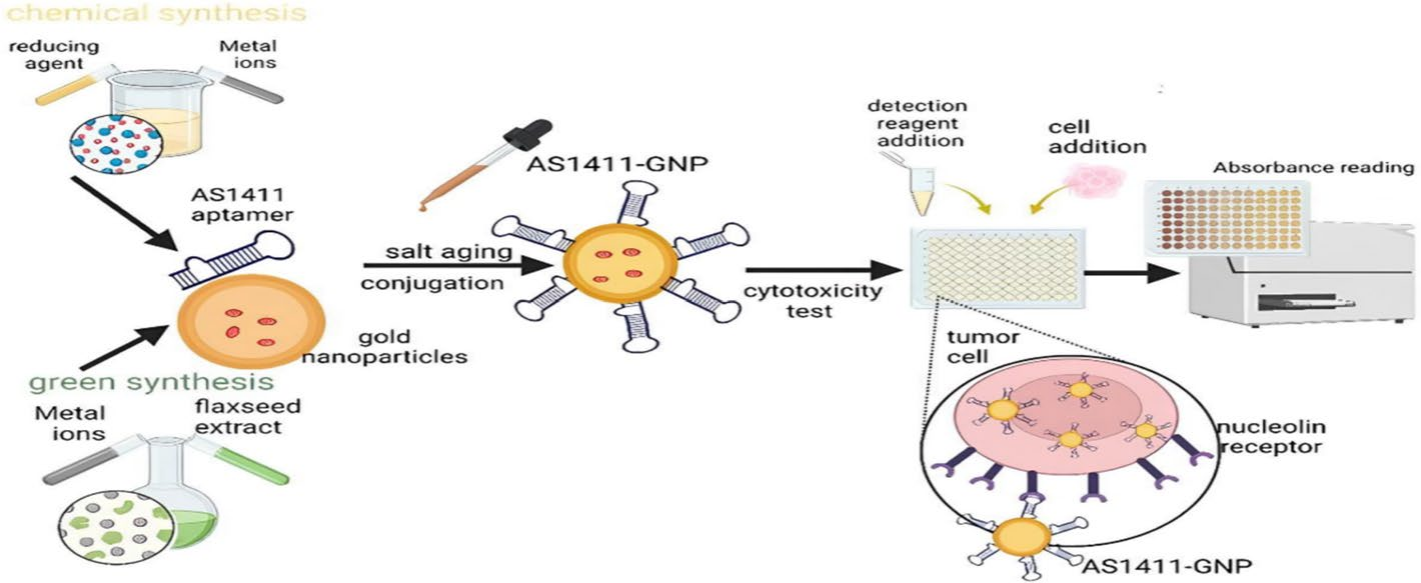
The workflow conducted in this study

Herein, we computationally validated the potential use of AS1411 aptamer in targeting the NCL receptor overexpressed on breast cancer cells. Furthermore, we experimentally investigated and compared their use in conjugation with both chemically and green synthesized gold nanoparticles for the selection of a better targeting therapeutic system for breast cancer cells with minimal effect on normal cells (Fig. 1).

## Materials and Methods

### Molecular Docking and Modeling of Aptamer AS1411 with NCL Receptor

#### Protein and Aptamer Structure Selection

The three-dimensional structures of human NCL and the AS1411 aptamer were retrieved from the RCSB Protein Data Bank (PDB) using PDB IDs: 2KRR and 4U5M, respectively. Among the 20 refined structures available for the NCL, model 9 was selected highlighting the best binding mode according to a study conducted by [11] for further analysis.

#### Molecular Docking

Molecular docking was performed to investigate the interaction between AS1411 and NCL using the Haddock web server [23]. Docking files were prepared by removal of water, and any interacting molecules followed addition of polar hydrogen atoms to optimize the structures for the subsequent molecular docking. The resultant predicted docking poses were later analyzed using BIOVIA Discovery Studio [24].

#### MD Simulation and Free Energy Calculations

The NCL-AS1411 complex, derived from the docking results, was subjected to molecular dynamics (MD) simulation using Gromacs 2023.2 software [25], employing the CHARMM36-Jul 2022—ff force field [26]. The complex was positioned within a cubic box with dimensions of 90 × 90 × 90 Å, and the TIP3P water model was used for solvation. To maintain electrical neutrality, potassium ions (K^+^) were added to achieve a concentration of 150 mM. The MD simulation comprised several key steps. First, a system minimization was performed over 1162 steps, resulting in a potential energy of − 8.87e + 05. This was followed by two stages of equilibration: the NVT ensemble as the initial stage and, subsequently, the NPT ensemble. Each equilibration stage spanned 5 ns, ensuring the system reached the desired physiological simulation conditions of 310 K for temperature and 1 bar for pressure [27, 28]. Temperature and pressure stability were maintained using the velocity-rescale modified Berendsen thermostat and Parrinello-Rahman pressure coupling method, respectively [29]. Finally, the MD simulation production phase was conducted at 310 K and 1 bar for a total duration of 50 ns. To determine free energy values, the MM/GBSA (molecular mechanics/generalized born surface area) method was applied to the trajectory files generated from the MD simulations, utilizing the GMX_MMGBSA tool [30]. Additionally, the MD results were thoroughly analyzed through root mean square deviation (RMSD) and root mean square fluctuation (RMSF) calculations. Graphical representation of the findings was accomplished using Xmgrace software [31].

### Chemical Synthesis of GNPs

GNPs were synthesized using the Turkevich method [32], as previously reported. Briefly, tetrachloroauric acid trihydrate HAuCl4·3H_2_O (Sigma-Aldrich, Germany) was dissolved in 50 ml of deionized water and brought to boil. Subsequently, tri-sodium citrate salt dehydrates (Topchem, U.S.) were then added under constant stirring as a reducing agent, maintaining an 11:1 molar ratio. The reaction proceeded until the distinctive burgundy-red color was observed indicating GNP formation. Afterwards, the reaction mixture was allowed to cool to room temperature, washed, and rehydrated in deionized water.

### Green Synthesis of Flaxseed Extract-Stabilized GNP Nanoparticles (Fs-GNPs)

The extraction of reducing agents and stabilizing components from flaxseeds was carried out using ethanol. Initially, 5 g of flaxseed extract was prepared as mentioned with minor modifications [33]. Briefly, the flaxseeds were cleaned, dried, and finely ground into powder. Subsequently, the flaxseed powder was immersed in 70% ethanol, homogenized, and incubated overnight. After the incubation period, the extract was filtered to remove any solid residues. The filtrate was then evaporated using a rotary evaporator, maintaining a controlled temperature of 40 °C. The resulting extract was rehydrated in deionized water and served a dual role as a reducing and stabilizing agent in the synthesis of the Fs-GNPs.

Fs-GNPs were prepared as follows: 5 mL of tetrachloroauric acid trihydrate solution was added to 5 mL of the ethanol extract. This mixture was placed in a shaking incubator at 40 °C until the distinctive deep burgundy color was observed, indicating the reduction of gold ions and the formation of Fs-GNPs. The solution was then washed and rehydrated in deionized water.

### Conjugation of Nanoparticles with AS1411 Aptamer

The thiolated AS1411 aptamer (5′ SH-GGTGGTGGTGGTTGTGGTGGTGGTGG 3′) was procured in its lyophilized form (Macrogen, South Korea) and reconstituted in DNase/RNase-free water to attain a concentration of 100 micromolar. In preparation for GNP and Fs-GNP conjugation, AS1411 aptamer molecules underwent reduction by the addition of a 100-fold excess of tris(2-carboxyethyl) phosphine (TCEP) in a V/V ratio equating to that of the aptamer. This mixture was then incubated in darkness for 1 h at room temperature, followed by heating at 95 °C for 5 min, and immediately cooled on ice.

Reduced aptamers were introduced to the nanoparticles in a molar ratio of 200:1. A solution containing 0.01% sodium dodecyl sulfate (SDS) (Sigma Aldrich, Germany) and 0.1 M phosphate buffer (PB) was added to the mixture, and the resultant composite was incubated for 20 min at ambient temperature. A salt aging process was performed [34] with minor modifications. Sodium chloride (NaCl) (SERVA, Germany) was gradually added until reaching final concentrations of 0.7 M for GNPs and 0.3 M for Fs-GNPs. To maintain the stability of the nanoparticles and prevent aggregation, between each NaCl addition, solutions were vortexed and ultrasonicated for 10 s to ensure complete dispersion allowing the samples to stabilize. The reaction was then incubated overnight at room temperature, followed by centrifugation at 15,000 rpm and 4 °C for a duration of 30 min for the removal of unreacted aptamers. The conjugates were then washed and re-suspended in PBS.

An alternate conjugation method was used for Fs-GNPs, a one-step conjugation approach, in attempting conjugation enhancement. Reduced aptamer molecules were combined with tetrachloroauric acid trihydrate solution in a 200:1 ratio. Following a vertexing and a 10-min incubation at room temperature with continuous shaking, the flaxseed extract was added to the mixture and incubated at room temperature until the appearance of a burgundy-red color.

### Characterization of Naked and Conjugated Nanoparticles

Characterization of the nanoparticles and their conjugates was undertaken through a battery of analytical techniques. Ultraviolet–visible (UV–VIS) spectroscopy was employed to ascertain the absorption peaks within the 400–600-nm wavelength range. The concentrations of these entities were determined via Beer-Lambert’s law, employing the corresponding extinction coefficients [35].

Hydrodynamic diameters, polydispersity indexes (PDIs), and zeta potentials were meticulously measured via dynamic light scattering (DLS) utilizing a Zetasizer instrument (Microtrac, Japan). Further evaluation of morphology and size was carried out via transmission electron microscopy (TEM) (JEOL, JEM-2100, Tokyo, Japan).

The electrophoretic gel migration properties of the nanoparticle conjugates were assessed. For the quantification of bound aptamers, conjugates were incubated overnight with 50 mM of dithiothreitol (DTT) (Sigma Aldrich, Germany). Subsequently, samples were subjected to centrifugation, and the supernatants containing free aptamers were quantified using UV–Visible spectrophotometry.

### In Vitro Analysis

To ascertain the cytotoxicity and therapeutic efficiency of the naked nanoparticles and their conjugates, a study was conducted on MCF-7 human breast cancer adenocarcinoma cells and WI-38 human lung fibroblast cells cell lines supplied by Vacsera (Giza, Egypt). The cells were cultured in specific media in 96-well tissue culture plates, with an approximate density of 10^5 cells per well. The culture medium contained fetal bovine serum (FBS) (Biowest, France) with a final concentration of 10%. Subsequently, GNPs, AS1411-GNPs, Fs-GNPs, and AS1411-Fs-GNPs were added in triplicate at concentrations ranging from 2.5 to 100 µg/ml. The plates were incubated at 37 °C with 5% CO_2_ for 24 h.

The MTT (3-[4, 5-dimethylthiazol-2-yl]−2, 5-diphenyltetrazolium bromide) assay was employed to evaluate cell viability. Following incubation, MTT solution was added and incubated for 4 h at 37 °C, 5% CO_2_ in the dark. Subsequently, isopropanol was added to each well, followed by thorough mixing. The plates were read in a UV–VIS spectrophotometer at 570 nm, with a background wavelength set at 630 nm. Cell viability was determined by subtracting the background absorbance at 630 nm from the absorbance of the formazan signal at 570 nm. This calculation yielded an absorbance value that directly correlated with the number of viable cells [36]. All nanoparticles were tested in triplicates.

### ROS Measurements

DCFDA-Cellular ROS assay Kit (Abcam, UK) was used to assess and quantify the cellular ROS in MCF-7 cells according to the kits’ instructions. The kit utilizes a cell-permeable 2′,7′-dichlorofluorescein diacetate, a non-fluorescence substance, which can be oxidized by intracellular ROS resulting in fluorescence emission. ROS was measured after treating 10^5 MCF-7 cells with IC_50_ concentrations of AS1411-GNPs and AS1411-Fs-GNPs for 24 h. Fluorescence reading was recorded at excitation/emission spectra of 485 nm/535 nm using a microplate fluorescent reader after 30-min incubation with the DCFDA solution in the dark at 37 °C with 5% CO_2_. Appropriate controls including untreated cells, cell culture media with DCFDA solution only, and TBHP (positive control) were used. The experiment was conducted in duplicate.

## Results

### Post-Docking Analysis

**Fig. 2:**
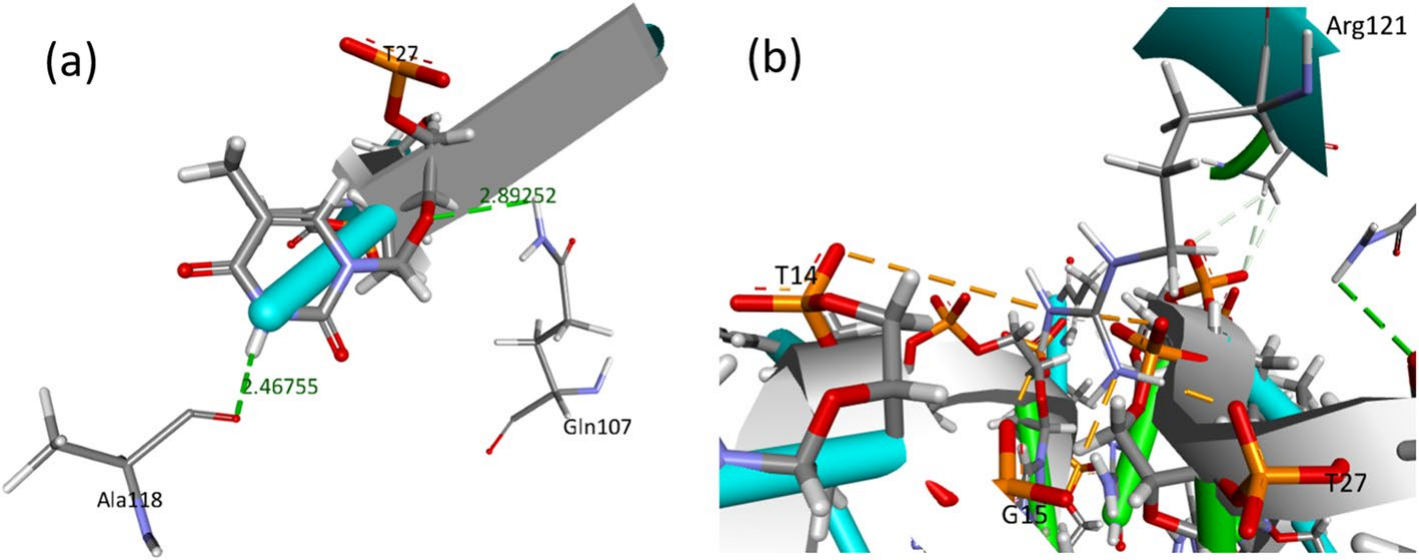
**a** Bonding interaction between human NCL and AS1411 aptamer formed 2 hydrogen bonds by nucleotide DT 27 with both ALA 118 and GLN 107 amino acids. **b** Another type of interaction the salt bridge interaction formed by nucleotides DT 27, DG 15, and DT 14 with ARG 121 amino acid

The undertaken post-docking analysis evaluated the quality of the AS1411 aptamer binding poses with the NCL. Among the Top 10 ranked clusters, cluster 2 had the most promising binding configuration with a remarkable docking score of − 149 ± 2.6. The selection of this pose was based on a meticulous assessment of interacting residues and the bonds formed between them, as visually represented in Fig. 2a, b. Specifically, the analysis unveiled a critical hydrogen bond with a bond length of 2.93 angstroms, establishing a strong interaction between ALA 118, a crucial residue positioned within the NCL binding site, and nucleotide DT 27 of the AS1411 aptamer. This hydrogen bond contributes significantly to the stability of the binding complex. Furthermore, a salt bridge was identified between ARG121 and DT 27, further enhancing the binding affinity between AS1411 and the NCL. Additionally, two salt bridges involving nucleotide DG15 were observed, highlighting the intricate nature of the binding interactions within this pose.

### Molecular Dynamics and Free Energy Calculation

**Fig. 3:**
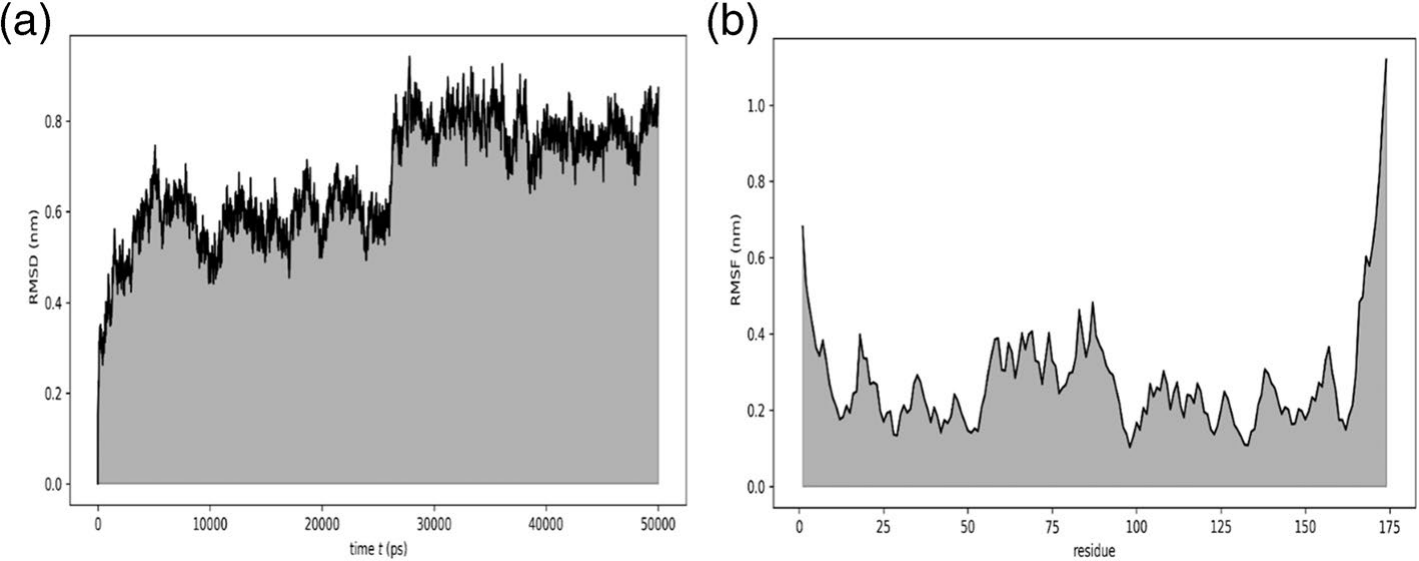
Illustrate **a** RMSD and **b** RMSF of the NCL-AS1411 complex extracted through 50-ns MD simulation

Subsequently, the chosen binding pose was subjected to MD simulation to gain insights into the dynamic behavior of the AS1411 aptamer-NCL complex over time. The analysis of RMSD and RMSF, as depicted in Fig. 3, provided valuable information regarding the stability and fluctuations of the system during the simulation. The results demonstrated that the complex maintained a stable behavior throughout the simulation period. Furthermore, the calculation of free energy using the MM/GBSA method yielded a highly negative value of − 102.96 kcal/mol indicating a robust and tightly bound interaction between the AS1411 and the NCL, emphasizing the favorable thermodynamics of the binding.

### Characterization of Naked GNPs and Fs-GNPs

**Fig. 4:**
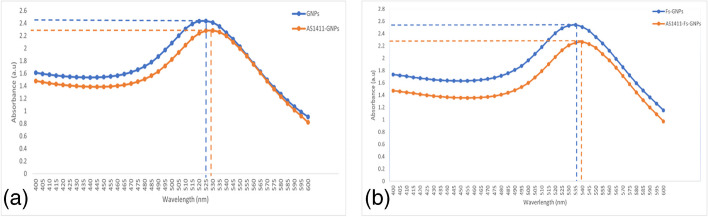
UV–visible spectroscopy analysis of GNPs. Graph (**a**) shows absorbance peaks for GNPs and AS1411-GNPs at 525 and 528 nm, respectively. Graph (**b**) shows absorbance peaks for Fs-GNPs and AS1411-Fs-GNPs at 536 and 539 nm, respectively. The observed peak shifts for both conjugates compared to their unconjugated counterparts indicate successful aptamer conjugation

The synthesis of GNPs nanoparticles was conducted via both chemical and green methods, the formation of red burgundy coloration is indicative of gold ion reduction and successful formation of the nanoparticles. DLS analysis indicated an average particle size of 26.16 nm and 33.2 nm with an average negative charge of 23.9 and 28.2 mV for GNPs and Fs-GNPs, respectively. Results from the UV–visible spectroscopy were comparable to that of DLS in terms of size. GNPs illustrated absorbance peaks at 525 nm, indicative of their characteristic size of approximately 20 nm. Fs-GNPs with a distinct absorbance peak at 536 nm suggest a larger size of approximately 40 nm illustrated in Fig. 4a and b.

**Fig. 5:**
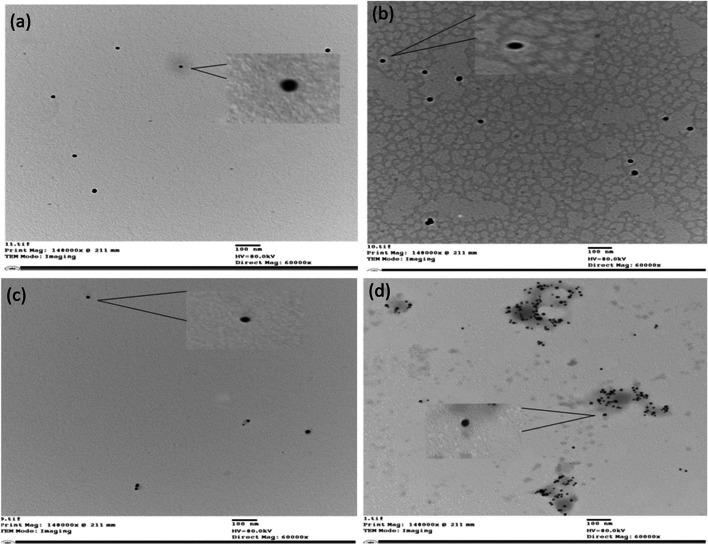
TEM images illustrating **a** GNPs, **b** AS1411-GNPs, **c** Fs-GNPs, and **d** AS1411-Fs-GNPs. All images are shown at a scale of 100 nm

Further insights into the size of the nanoparticles were garnered through TEM analysis shown in Fig. 5a and c. The chemical method yielded monodispersed GNPs with an average diameter of 21 ± 1.3 nm, showing their well-defined spherical nature. The Fs-GNPs also showed monodispersed particles with an average size of 12.8 ± 1.87 nm.

### Characterization of the Conjugated Nanoparticles

To confirm the successfulness of GNP and Fs-GNP conjugation with the AS1411 aptamer, a series of characterizations were performed, and their results were compared to that of the naked nanoparticles. DLS analysis showed an average size of 31.2 nm and 41.1 nm and an average negative charge of 27.5 and 34.1 mV for AS1411-GNPs and AS1411-Fs-GNPs, respectively. The UV–Vis spectrophotometer results showed an absorbance peak at 528 and 539 nm for AS1411-GNPs and AS1411-Fs-GNPs, respectively. TEM results showed well-dispersed nanoparticles of size 24 ± 1.93 and 17.2 ± 1.81 for AS1411-GNPs and AS1411-Fs-GNPs, respectively (Fig. 5b and d).

Regarding the one-step synthesis and conjugation method, UV–Visible spectrophotometer results showed a peak at 550 nm which corresponds to a size of approximately 70 nm. DLS results indicated an average particle size of 47.6 nm with an average negative charge of 32.9 mV.

Further characterization of the conjugate systems involved the incubation with DTT, which detaches the aptamers from the nanoparticles’ surface. Subsequent measurement of the concentration of detached aptamers approved the conjugation of AS1411 aptamer to both GNPs and Fs-GNPs compared to the negative control of their naked compartment. AS1411-GNPs displayed a slightly higher aptamer concentration attached to their GNPs compared to the Fs-GNP counterparts (S1 in supplementary data). However, this was not the case for the one-step synthesis and conjugation method as there was no detectable free aptamers after the DTT step, indicating conjugation failure. Consequently, this conjugate was eliminated from TEM analysis and the subsequent in vitro testing.

Moreover, a gel shift assay was employed as a confirmatory technique for assessing the conjugation process. Both AS1411-GNPs and AS1411-Fs-GNPs exhibited different migration patterns in the gel comparing each one to its naked counterpart, confirming the successful conjugation of AS1411 to both nanoparticles (S1 in supplementary data).

### Cytotoxicity and Therapeutic Efficiency Assessment Using MTT Assay

**Fig. 6:**
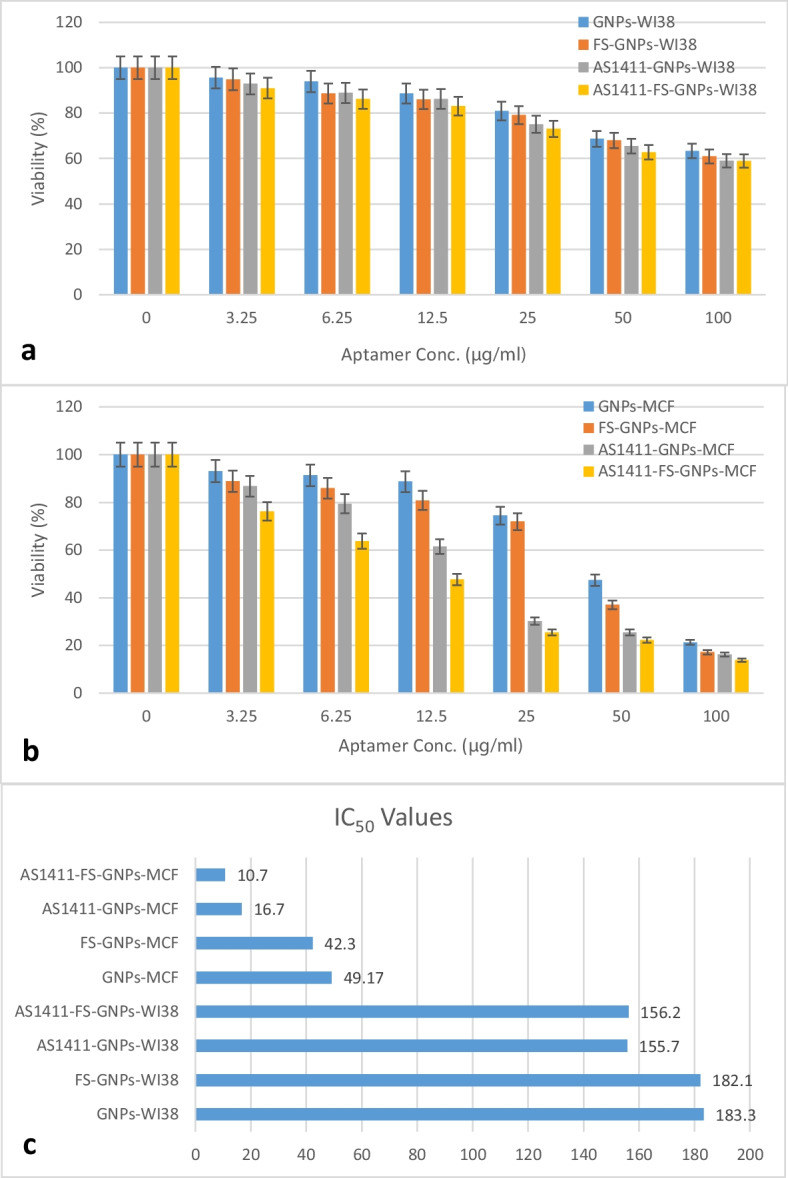
Comparative cytotoxicity of different concentration of GNPs, Fs-GNPs, AS1411-GNPs, and AS1411-Fs-GNPs on WI-38 (**a**) and MCF-7 (**b**). Cells as well as comparing of the detected IC_50_ values (**c**)

The cytotoxicity of naked nanoparticles and the conjugates was evaluated using the MTT assay on both normal human lung fibroblasts (WI-38) and breast cancer cells (MCF-7). This assay provides a reliable measure of cell viability by assessing the metabolic activity of cells, which is indicative of their proliferation potential [36]. The absorbance values at 570 nm obtained from the MTT assay were used to determine cell viability, and the results are presented as a percentage of viable cells compared to the untreated control (Fig. 6a and b). The IC_50_ values were calculated and presented in Fig. 6c.

When exposed to different concentrations of the nanoparticles, WI-38 cells exhibited a relatively high tolerance of > 50% viability to all tested nanoparticles even at the highest tested concentration of 100 µg/mL. On the contrary, the cell viability percentages of MCF-7 cells decreased drastically with the increase of the tested nanoparticle concentrations especially for the AS1411-GNPs and AS1411-Fs-GNPs showing increased susceptibility to the treatments, reflecting their greater sensitivity.

The IC_50_, which represents the concentration required for a 50% reduction in cell viability, was notably comparable and high with values of 183.3 and 182.1 µg/mL for the GNPs and Fs-GNPs when tested on WI-38 cells. However, their conjugated form, AS1411-GNPs and AS1411-Fs-GNPs, displayed lower values of 155.7 and 156.2 µg/mL, respectively.

In contrast to WI-38 cells, IC_50_ values were much lower with the MCF-7 for all four tested nanoparticles. For GNPs and Fs-GNPs, IC_50_ values were 49.17 and 42.3 µg/mL, respectively, showing greater impact and higher toxicity of the Fs-GNPs on the cells. AS1411-GNPs and AS1411-Fs-GNPs demonstrated significant IC_50_ values of 16.7 and 10.7 µg/mL, respectively, emphasizing the higher potential of AS1411-Fs-GNPs in inhibiting MCF-7 cells proliferation.

### ROS Generation in Treated MCF-7 Cells

**Fig. 7:**
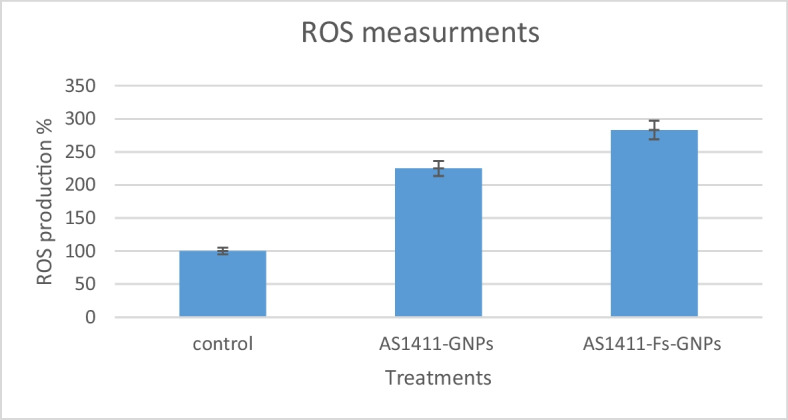
ROS activity of MCF-7 treated cells with AS1411-Fs-GNPs and AS1411-GNPs compared to untreated cells

DCFDA-cellular ROS detection assay was utilized to investigate the role of ROS in the anticancer effects of AS1411-GNPs and AS1411-Fs-GNPs on MCF-7 cells. Overproduction of cellular ROS can cause harm to many cellular components including proteins, nucleic acids, membranes, and organelles triggering cell apoptosis. It is one of the pathways by which the aptamer-conjugated nanoparticles could induce cytotoxicity. The increase in fluorescent intensity is directly proportional to the ROS generation. After subtracting the background signal for all samples, the untreated cell control fluorescent intensity was set to 100% presenting baseline ROS activity. Our results showed that the AS1411-Fs-GNPs induced more ROS generation than AS1411-GNPs. In the case of AS1411-Fs-GNP-treated cells, ROS increased 2.8-fold while it increased 2.2-fold for the AS1411-GNP-treated cells more than the ROS of untreated cells (Fig. 7). In both cases, the increase in ROS production compared to the untreated cells is a critical indicator of cellular stress which can eventually result in cell death. These results emphasize the potential mechanisms by which AS1411-GNPs and AS1411-Fs-GNPs exert their cytotoxic effects on the MCF-7 cells. It also highlights the potential greater effect of AS1411-Fs-GNPs on cancerous cells.

## Discussion

The field of cancer treatment is rapidly evolving, with targeted therapeutic systems emerging as a promising avenue for more effective and precise interventions. These systems offer the potential to selectively target and eliminate cancer cells while minimizing harm to healthy ones [37].

Our study focused on the design and assessment of a targeted therapy system for breast cancer. It extends four interconnected pathways: first, an in silico investigation including molecular docking and dynamics ensuring stable and strong interaction between AS1411 aptamer and the NCL receptor; second, the synthesis of gold nanoparticles using green and chemical methods; third, the development of conjugated systems composed of the synthesized nanoparticles and AS1411 aptamer; and fourth, comparing between the bare nanoparticles and their conjugates in terms of effect on normal (WI-38) and cancer (MCF-7) cell lines.

Recently, GNPs have garnered significant attention for their unique physicochemical properties that make them ideal candidates for various applications in cancer treatment. Biologically active compounds extracted from natural sources and used in green synthesis of GNPs could exert powerful anticancer effects. The effectiveness and cellular impacts of biosynthesized GNPs are dependent on the biological extract utilized in the synthesis process, although it has been shown that many of these nanoparticles have anticancer activity [38]. We delved into testing this theory by employing green and chemically synthesized GNPs on cancer cells as well as testing their usage in a conjugated system with the targeting molecule, AS1411 aptamer, which would enhance their treatment ability towards cancer cells and consequently their usage as an efficient target therapy.

In the preparation of green synthesized GNPs, ethanol-based extract from flaxseeds was used as an environmentally friendly approach with significant implications for cancer treatment [33], acting as both reducing and stabilizing agents. Furthermore, the bioactive compounds including lignans, phenolic acids, and polysaccharides, which can serve as effective reducing and stabilizing agents for nanoparticle synthesis [20] present in the flaxseed extract offer potential benefits to normal cells reinforcing.

Our green synthesized GNPs were compared to the well-established chemical synthesized GNPs by the Turkish method, as it allowed us to establish a strong comparative framework with our green synthesis approach especially since that has extensively been used and studied before in the treatment of different types of cancer cells [39–41]. In addition, several studies have shown that AS1411 aptamer made it easier for different nanoparticles to get into cancer cells. We investigated their conjugation to the chemical and green synthesized gold nanoparticles and also their impact on normal and cancer cells [42].

In our investigation, comparing between GNPs, Fs-GNPs, and their conjugate counterparts, we noticed that the UV–Visible spectra peaks shifted from 525 to 528 and 536 to 539 nm for the AS1411-GNPs and AS1411-Fs-GNPs, respectively. This shift in the wavelength peak demonstrated the specific binding of AS1411 to the GNPs and Fs-GNPs. As well, the DLS results showed an increase in the average size from 26.16 to 31.2 and 33.2 to 41.1 nm for AS1411-GNPs and AS1411-Fs-GNPs, respectively. Additionally, we noticed an increase in the average charge from 23.9 to 27.5 and from 28.2 to 34.1 mV for AS1411-GNPs and AS1411-Fs-GNPs, respectively. Moreover, in the gel shift assay, GNPs and Fs-GNPs showed a different migration pattern than their conjugated counterparts. All tests indicated the successful conjugation of AS1411 aptamer to both: GNPs and Fs-GNPs. Nevertheless, the conjugation was further confirmed through the increase of size and presence of a surrounding halo “corona” in the conjugated nanoparticles and with the DTT solution where the detached aptamers from the gold nanoparticles’ surfaces were quantified indicating successful conjugation.

Following the characterization and evaluation of our synthesized nanoparticles, cytotoxicity tests were conducted on normal and cancer cells exposed to the four treatments: GNPs, AS1411-GNP Fs-GNPs, and AS1411-Fs-GNPs. Cytotoxicity assessment was performed to evaluate the impact of the different nanoparticle treatments on the proliferation of MCF-7 and WI-38 cells. Cell viability was assessed across a concentration range of 0 to 100 μg/mL for each treatment, and the half-maximal inhibitory concentration (IC_50_) values were determined, providing insights into their cytotoxicity profiles.

For normal lung cells (WI-38), the cytotoxicity profiles of the nanoparticle treatments exhibited a common trend. At lower concentrations, ranging from 1.25 to 25 µg/mL, all treatments displayed minimal cytotoxicity with cell viabilities above 70%. This finding aligns with the biocompatibility of the nanoparticles with normal cells at lower doses. However, as the nanoparticle concentrations increased, a gradual decline in cell viability was observed, indicating a dose-dependent cytotoxic effect. Significantly, the IC_50_ values, representing the concentration required to inhibit 50% of cell growth, ranged from 155.7 to 183.3 µg/mL for the WI-38 cells. We noticed an increase in the toxicity of the AS1411 conjugated nanoparticle in contrast to their naked counterparts. This observation may be due to the presence of a minute amount of NCL receptor on the surface of the normal cells which enhanced the uptake of the nanoparticle and exerted their toxic effect towards these cells [12].

Conversely, when we assessed the impact of nanoparticle treatments on cancerous MCF-7 cells, the treatments demonstrated substantially higher cytotoxic effect leading to lower proliferation than WI-38 cells. The determined IC_50_ values were between 10.7 and 49.17 µg/ml. Remarkably, the AS1411 conjugated nanoparticles outperformed their naked counterparts displaying lower IC_50_ values of 16.7 and 10.7 µg/mL for AS1411-GNPs and AS1411-Fs-GNPs, respectively. Indicating their superior inhibitory efficacy and emphasizing the role of AS1411 aptamer in enhancing the uptake of the treatment through binding to the overexpressed NCL on the MCF-7 cells. Fs-GNPs and AS1411-Fs-GNPs showed lower IC_50_ values of 42.3 and 10.7 µg/mL compared to the 49.17 and 16.7 µg/mL for GNPs and AS1411-GNPs, respectively. At the concentration of 12.5 µg/mL, AS1411-GNPs and AS1411-Fs-GNPs showed < 60% and < 50% of MCF-7 viability, respectively, while both preserved > 80% WI-38 viability. At higher a concentration, 25 µg/mL, AS1411-GNPs and AS1411-Fs-GNPs showed about 30% and 25% of MCF-7 viability, respectively, while both preserved > 70% WI-38 viability. Although lower concentrations of the conjugated nanoparticles preserved a higher viability percentage of WI-38, it demonstrated less impact (< 50%) on the MCF-7 cells. Thus, our finding suggests the potential use of AS1411-Fs-GNPs at the concentration range of 12.5 to 25 µg/mL as a therapeutic agent against breast cancer cells to preserve minimal healthy cell damage in further studies. Furthermore, the AS1411-Fs-GNPs induced more ROS generation in the treated MCF-7 cells than the AS1411-GNPs resulting in more cellular stress leading to cellular death. Additional investigations including precise mode of action and in vivo impact should be conducted to further validate AS1411-Fs-GNP potential use in breast cancer treatment.

Our findings align with previous research, highlighting the potential of aptamer-conjugated nanoparticles for targeted cancer therapy [43, 44]. Moreover, they agreed with previous investigations that have emphasized the pivotal role of the AS1411 aptamer in enhancing the selective uptake of GNPs by breast cancer cells, particularly MCF-7 and MDA-MB-231 cells with an IC_50_ equivalent to < 100 nM [45]. Other prior studies also highlighted the therapeutic potential of AS1411/GNPs in breast cancer treatment at comparable concentrations to our results [42, 46, 47]. Aptamer selectivity holds significant promise for targeted cancer therapy, facilitating the preferential accumulation of therapeutic agents within cancerous cells while sparing normal tissues. The high affinity of the AS1411 aptamer for nucleolin plays a pivotal role in mediating this selective uptake. In a further study, the antiproliferative activity of GNPs was rigorously examined by assessing their ability to inhibit the proliferation of MCF-7 breast cancer cells. This outcome highlights the significance of GNPs in sensitizing breast cancer cells to therapeutic interventions, offering a promising opportunity for targeted cancer therapy.

In other studies, green synthesized GNPs from Ziziphus spina-Christi showed enhanced reduction in cancer cell viability. These findings indicate the potential of green GNPs as an effective breast cancer therapy [18]. However, the cytotoxicity of green synthesized GNPs alone showed limited impact on MCF-7 cells, suggesting that combining them with NIR irradiation is essential to unlocking their full therapeutic potential. This observation agreed with our result where the Fs-GNPs alone showed an IC_50_ value of 42.3 µg/mL while the AS1411-Fs-GNPs showed an IC_50_ value of 10.7 µg/mL. The AS1411 aptamer seems to unlock the full potential of our therapeutic system.

These findings underscore the potentiation of AS1411’s antiproliferative activity when conjugated to GNPs, signifying the remarkable synergy between the aptamer and GNPs in inhibiting breast cancer cell growth [47]. Furthermore, additional previous work has observed the selectivity of a specific cancer cell, as opposed to non-malignant cells, further highlighting the preferential drug uptake and delivery of AS1411-based nanoparticles to cancer cells [48]. This selectivity is paramount in minimizing off-target effects and preserving the viability of healthy cells, a principle that aligns with our research in the context of breast cancer. However, while our study provides preliminary insights into the potential use of green-synthesized-gold-nanoparticles-aptamer conjugate for targeted breast cancer therapy, further research including different pathway analysis, on-target/off-target effect determination, and in vivo studies are necessary to validate our findings and determine the therapeutic potential and biocompatibility of this conjugate.

## Conclusion

Our study explored the potential of AS1411-conjugated to GNPs and Fs-GNPs for targeted breast cancer therapy. Through a comprehensive approach incorporating molecular docking and modeling, GNP and Fs-GNP synthesis, AS1411 aptamer conjugation, characterization and validation of treatments, and WI-38 and MCF-7 cells cytotoxicity assessments, we have generated valuable insights with promising implications for cancer treatment. Our molecular docking incorporated with molecular dynamics simulation studies have demonstrated a strong binding affinity between the AS1411 aptamer and the nucleolin receptor highlighting the aptamer’s ability to specifically target cancer cells crucial for reducing side effects and improving treatment precision.

AS1411 aptamer has been successfully conjugated with GNPs and Fs-GNPs and was confirmed through various characterization techniques including DLS, TEM, gel shift, and DTT assays. Our cytotoxicity assessment revealed an enhanced therapeutic efficiency of AS1411-GNPs and AS1411-Fs-GNPs towards MCF-7 cells compared to GNPs and Fs-GNPs with the edge for AS1411-Fs-GNPs with IC_50_ value of 10.7 µg/mL. Our findings suggested the potential use of AS1411-Fs-GNPs at a concertation range of 12.5 to 25 µg/mL to keep a high viability of 70–80% for normal cells while reducing > 50% of the cancer cells. Yet, further studies to determine the on-target/ off-target effect as well as possible pathways of the AS1411-Fs-GNPs should be conducted.

## Supplementary Information

Below is the link to the electronic supplementary material.

Supplementary file1 (PDF 229 KB)

## Data Availability

Additional data can be found in the supplementary material folder.

## Abbreviations

*GNPs*: Chemically synthesized gold nanoparticles
*Fs-GNPs*: Green synthesized gold nanoparticles
*AS1411-GNPs*: Chemical conjugate
*AS1411-Fs-GNPs*: Green conjugate
*TEM*: Transmission electron microscopy
*DLS*: Dynamic light scattering
*UV-Vis*: UV-visible spectrophotometer
*Non-SCC*: Non-stem cancer cells
*CSC*: Cancer stem cells
*IC50*: Inhibitory concentration
*DMEM*: Eagle’s minimum essential medium
*PDI*: Polydispersity indexes
*DTT*: Dithiothreitol
*NCL*: Nucleolin
*EPR*: Permeability and retention effect
*ROS*: Reactive oxygen species
*RPMI 1640*: Roswell Park Memorial Institute 1640 Medium
*MTT*: 3-[4, 5-Dimethylthiazol-2-yl]-2, 5-diphenyltetrazolium bromide
*PBS*: Phosphate buffer saline
*PB*: Phosphate buffer
*SDS*: Sodium dodecyl sulfate
*DCFDA*: 2′,7′-Dichlorofluorescin diacetate
