# Supplementary material for: Comparative Analysis of Aptamer-Conjugated Chemical and Green Synthesized Gold Nanoparticles for Targeted Therapy in MCF-7 Cancer Cells
Source: Appl Biochem Biotechnol. 2024 Nov 27;197(3):1678–95. doi: 10.1007/s12010-024-05091-2 (PMC11953193; doi:10.1007/s12010-024-05091-2)
Supplement: Supplementary file 1 — Supplementary file1 (PDF 229 KB) [file 12010_2024_5091_MOESM1_ESM.pdf]

Comparative Analysis of Aptamer-Conjugated chemical and green synthesized Gold Nanoparticles for Targeted Therapy in MCF-7 Cancer Cells.

Mariam W. Helal [1], Mohanad M. Faried [1], Sohaila Mohammed Salah [1], Mazen Ashraf [1], Nada Nasser [1], Yasser Shawky [1], Sara Hamdy [1], Azza El Amir [1], Wajeet Nabil [2], Dalia M. El-Husseini [3].

[1] biotechnology department, Faculty of Science, Cairo University, Egypt
[2] Zoology Department, Cairo University, Egypt
[3] Nanomaterial research and synthesis unit, Animal Health Research Institute, Egypt.

Co-Author:
Dalia M. El-Husseini: dalia_biotech@yahoo.com

***S1** Illustrate the concentration of detached aptamers for conjugate systems after using their naked counterparts as blank.*

Sample	Aptamer concentration (ng/μl)
AS1411- Fs-GNPs	37.8
AS1411-GNPs	39.3
One pot	0.001

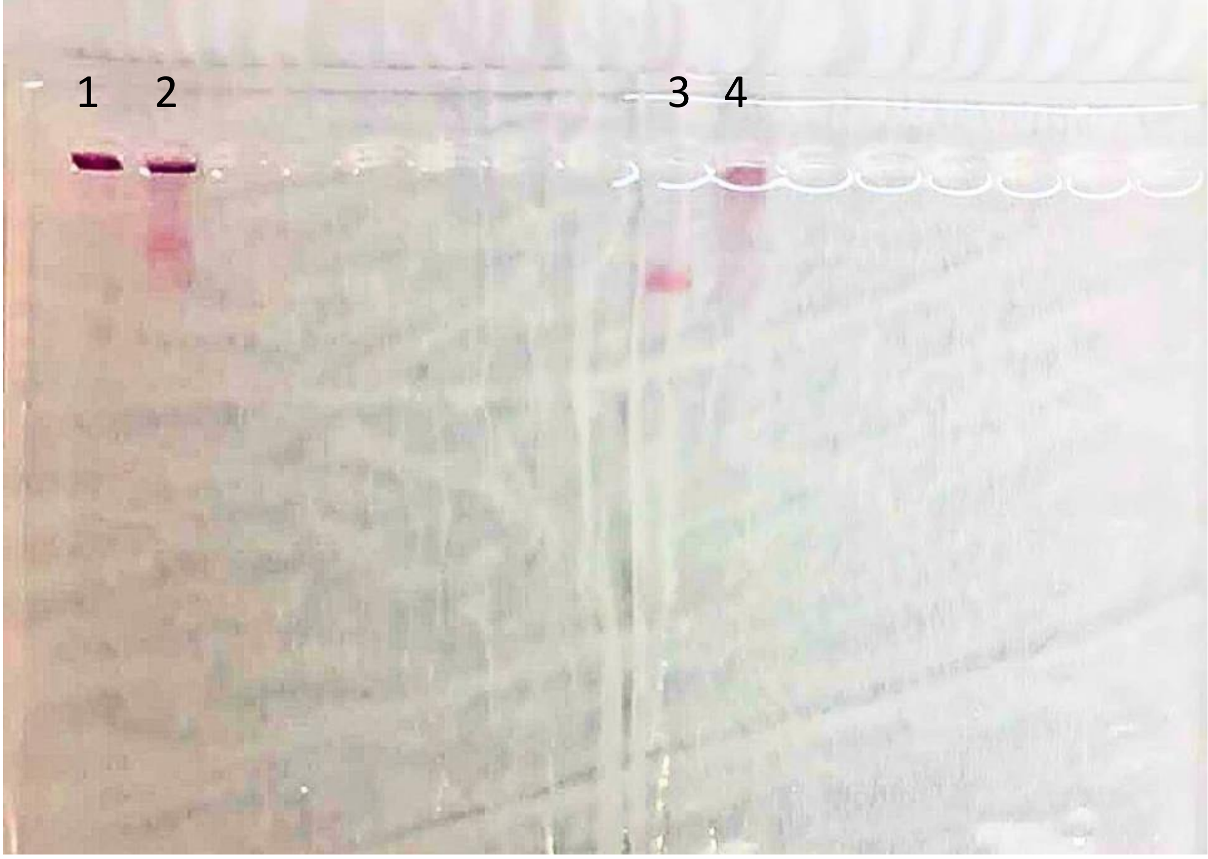

S2 Gel shift assay (1.5% agarose), lane 1 AS1411-Fs-GNPs, lane 2 Fs-GNPs, lane 3 the AS1411-GNPs, lane 4 GNPs.
